# Friendship Context Matters: Examining the Domain Specificity of Alcohol and Depression Socialization Among Adolescents

**DOI:** 10.1007/s10802-012-9625-8

**Published:** 2012-03-23

**Authors:** Matteo Giletta, Ron H. J. Scholte, Mitchell J. Prinstein, Rutger C. M. E. Engels, Emanuela Rabaglietti, William J. Burk

**Affiliations:** 1 Department of Psychology, University of Torino, via Verdi 10, 10124 Torino, Italy; 2 Behavioural Science Institute, Radboud University Nijmegen, Nijmegen, The Netherlands; 3 Department of Psychology, University of North Carolina, Chapel Hill, NC USA

**Keywords:** Peer influence, Friendship, Networks, Dyads, Depressive symptoms, Alcohol misuse

## Abstract

Driven by existing socialization theories, this study describes specific friendship contexts in which peer influence of alcohol misuse and depressive symptoms occurs. In the fall and spring of the school year, surveys were administered to 704 Italian adolescents (53 % male, *M*_age_ = 15.53) enrolled in Grades 9, 10 and 11. Different friendship contexts were distinguished based on two dimensions referring to the level (i.e., best friendships and friendship networks) and reciprocity (i.e., unilateral and reciprocal) of the relationships. Social network and dyadic analyses were applied in a complementary manner to estimate peer socialization effects across the different friendship contexts. Results showed that within friendship networks both male and female adolescents’ alcohol misuse was affected by friends’ alcohol misuse, regardless of whether the relationship was reciprocated or not. Conversely, peer socialization of depressive symptoms only emerged within very best friendship dyads of female adolescents. Findings suggest that the effects of peer socialization depend on the friendship context and specific types of behaviors. The theoretical and methodological implications of the findings are discussed.

It is well known that peers, and especially friends, play a central role in children’s and adolescents’ psychosocial development (Berndt 1982; Buhrmester 1990; Hartup 1989). Besides the positive effects of peer relationships on adolescent psychological adjustment, many studies have shown that peers may also impair adolescent development. Peers may affect adolescents’ behaviors and emotions through socialization processes, a phenomenon more generally referred to as peer influence (Prinstein and Dodge 2008). Socialization indicates the tendency of relationship partners (e.g., friends) to influence each other behaviors and therefore increase their similarity over time. This process must be distinguished from selection, which may also result in peer similarity, but refers to the tendency of adolescents to initiate relationships with peers who exhibit similar behaviors (Kandel 1978). Peer influence research is extremely important given that, even after accounting for selection processes, peer socialization has been reported to be a strikingly powerful and consistent phenomenon that occurs within a variety of friendship contexts (e.g., intimate best friendships as well as larger peer networks including all friendships) for a wide range of externalizing behaviors (e.g., aggression, delinquency, and substance use; Dishion et al. 1996; Sijtsema et al. 2010; Urberg et al. 2003; Vitaro et al. 2000) and internalizing behaviors (e.g., depressive symptoms, non-suicidal self-injury and social anxiety; Mercer and Derosier 2010; Prinstein et al. 2010; Van Zalk et al. 2011).

Yet, although prior work has provided strong evidence supporting peer socialization, it remains unclear whether socialization of different behaviors occurs across various friendship contexts (e.g., best friendships and friendship networks) or, conversely, whether socialization of certain behaviors exclusively pertains to certain types of friendship only. Indeed, most research in this area has relied on the preliminary but untested assumption that socialization processes may operate similarly for a wide variety of behaviors (Brechwald and Prinstein 2011); however this assumption rarely has been empirically examined. Understanding which friendships place adolescents at risk for socialization of which behaviors may be crucial to furthering our knowledge about adolescent peer influence. Thus, the present study examined peer influence related to two different domains, alcohol misuse and depressive symptoms, within various friendship contexts, reflecting different relationship levels (i.e., best friendships and multiple friendships within a peer network) and reciprocity (i.e., unilateral and reciprocal). Our choice to focus on alcohol misuse and depressive symptoms derived from three main reasons, including their increase and high prevalence during adolescence (e.g., Cole et al. 2002; Hankin et al. 1998; Johnston et al. 2008), the relevance of peer socialization in affecting their development, and the fact that different processes may be expected to underlie socialization of alcohol misuse and depressive symptoms (as detailed below).

## Peer Influence and Friendship Contexts: Existing Evidence

In this study two interrelated dimensions of friendship are considered: (a) the level of the friendship, here conceptualized as dyadic best friendships versus multiple overlapping friendships within a peer network, and (b) the reciprocity of friendship, that is, unilateral (i.e., one peer nominates the other who does not return the nomination) versus reciprocal (i.e., both peers mutually nominate each other as friend) friendships. Regarding the level of the friendship, most research has studied peer influence within dyadic friendships, with the aim to identify adolescents’ closest and most significant relationships. Here, usually adolescents’ very best friends, or the highest one in the rank of their top three best friends, are selected and examined as unique sources of influence. These prospective studies provided evidence for socialization effects in best friendships in relation to several behaviors, including alcohol use (e.g., Jaccard et al. 2005; Poelen et al. 2007; Popp et al. 2008) and depressive symptoms (e.g., Giletta et al. 2011; Prinstein 2007).

Other work has extended the analysis of peer influence from best friends’ dyads to the larger peer network, recognizing a major shortcoming of dyadic studies and emphasizing the importance of considering relations other than best friendships. Most adolescents have more than one friend and while best friends might represent a primary source of influence, characteristics and behaviors of all friends, as well as the norms established within peer groups, may contribute to socialization processes (Hartup 1993; Haynie 2001). Recent advances in data analysis techniques have provided the opportunity to evaluate a more complete and less selective picture of adolescent relationships by examining multiple overlapping friendships within larger peer networks (Carrington et al. 2005). The advantage of these methods includes the ability to account for relational and statistical dependencies in friendship networks, allowing researchers to examine all relationships among network participants simultaneously, not just non-overlapping best friendships. Further, these methods are capable of estimating the effects of peer socialization while accounting for numerous alternative explanations, such as homophilic peer selection (i.e., friendship selection based on behavioral similarities). This line of research has shown socialization effects within friendship networks regarding various domains, among which alcohol use (Kiuru et al. 2010; Knecht et al. 2011; Rabaglietti et al. 2011) and depressive symptoms (Kiuru et al. 2011; Mercer and Derosier 2010; Van Zalk et al. 2010).

These findings seem to suggest that peer influence is similar in best friendship dyads and peer networks, and in alcohol use and depressive symptoms. However, because friendship networks encompass several types of relations (e.g., dyadic and triadic, unilateral and reciprocal, same- and cross-gender), social network analyses do not directly allow to determine whether socialization processes actually occur in the peer networks at large or whether, on the contrary, network effects simply reflect the effects of only some dyadic relationships (e.g., best friend dyads). Thus, to accurately estimate the extent to which these two levels of friendship contribute to peer socialization, dyadic and social network approaches need to be combined. Few studies have tested socialization processes across different friendship levels, showing that both adolescents’ best friends and friend group independently contributed to peer influence in relation to smoking and drinking behaviors (Urberg 1992; Urberg et al. 1997). However, to date, no single study has simultaneously investigated peer influence related to different behavioral domains across different friendship levels.

Reciprocity represents another dimension of adolescent friendships (Newcomb and Bagwell 1995). Reciprocity is a dyadic attribute which describes the relationship between two individuals; therefore most of previous studies that examined the effect of reciprocity on peer socialization have focused on dyadic relationships, mainly best friendships. These studies showed that unilateral and reciprocal friendships may exert different influences (e.g., Bot et al. 2005b; Jaccard et al. 2005; Stevens and Prinstein 2005). Specifically, two hypotheses exist with respect to the role of reciprocity in friendships on peer socialization (Brechwald and Prinstein 2011; Dishion and Tipsord 2011). On the one hand, it has been suggested that reciprocal friendships, especially reciprocal best friend dyads, may provide a primary context for mutual influence, because within these relationships adolescents have the greatest opportunity to interact, to be intimate and specifically share their thoughts and personal feelings (e.g., Buhrmester 1990; Rose 2002). Evidence supporting this hypothesis has been found in relation to both peer socialization of alcohol use and depressive symptoms (e.g., Stevens and Prinstein 2005; Urberg et al. 2003).

In contrast, an alternative hypothesis suggests that peer influence may be more prominent within unilateral relationships. According to this idea, adolescents are more likely to change their behaviors or attitudes in order to conform to their desired friends and, therefore, increase their chance of establishing reciprocal and more intimate relationships with them. Previous studies supported this hypothesis, specifically with respect to peer socialization of substance use (i.e., alcohol and tobacco use) by showing that adolescents were more likely to adapt their behavior to the behavior of the peers they unilaterally choose as friends (Aloise-Young et al. 1994; Bot et al. 2005b; Jaccard et al. 2005). Research analyzing the role of friendship reciprocity in peer networks is scarce. Studies that do exist suggest that friendship reciprocity may not affect socialization processes at all, neither for alcohol use nor for depressive symptoms (Burk et al. 2012; Kiuru et al. 2011; Mercer and Derosier 2010; Mercken et al. 2012).

Thus, the role of reciprocity in promoting or inhibiting socialization processes within friendships remains unclear. Importantly, equivocal results may be due to issues regarding the behaviors that are investigated (i.e., alcohol use or depressive symptoms) and the level of the relationship in which reciprocity is examined (i.e., best friendship dyads or friendship networks). Here, we argued that the way with which friendship level and reciprocity affect peer socialization may vary as a function of the specific behavior which is the subject of influence (Hartup 2005).

## Peer Influence and Friendship Contexts: The Domain Specificity Hypothesis of Alcohol Misuse and Depressive Symptoms

The role of different contexts of friendship in socialization processes may be specific for each behavioral domain, for instance externalizing behaviors (e.g., alcohol use) as compared to emotional states (e.g., depressive symptoms) (Hartup 2005). Theoretical models that have been proposed to elucidate the processes underlying peer socialization (e.g., social learning theories, identity-based theories; for a review, see Brechwald and Prinstein 2011) have been applied mainly to the study of peer influence on externalizing behaviors, such as substance use (e.g., alcohol use) and delinquency. These models emphasize the functional values that such behaviors have within adolescent social relationships. That is, adolescents may be more inclined to model those peers’ behaviors that are reinforced in order to profit from the resulting objective or perceived social benefits, such as gaining social status (i.e., popularity) and approval from their peer group, or adhering to their group norms (e.g., Brechwald and Prinstein 2011; Cohen and Prinstein 2006). This may be the case for alcohol misuse as during adolescence drinking behavior may be associated with high social status and group acceptance (e.g., Allen et al. 2005; Mayeux et al. 2008). Thus, based on these theories, it is plausible that socialization of alcohol occurs across different friendship contexts, regardless of the level and reciprocity of the relation. For example, on the one hand, adolescents may conform to the behaviors they observe within their peer networks in order to achieve a higher social status within their group. On the other hand, within friend dyads, adolescents may be affected by their best friends’ behavior through specific dyadic interpersonal dynamics (i.e., deviancy training; Dishion et al. 1996). Hence, peer influence may indistinctly characterize unilateral as well as reciprocal friendships.

However, socialization of emotional states, in particular depressive symptoms, may depend on different mechanisms. According to the interpersonal theory of depression (Coyne 1976), depression contagion would occur as a consequence of the maladaptive interpersonal patterns (e.g., excessive reassurance seeking) that depressed individuals tend to exert in their dyadic relationships. These behaviors are likely to generate stress in the relational partners of depressed individuals, which consequently may be at risk to develop depressive symptoms themselves (Joiner and Timmons 2009). Specifically, these processes may operate within close and intimate relations because in these friendships, adolescents have many opportunities to interact and share their personal feelings and emotional states (e.g., Buhrmester 1990; Newcomb and Bagwell 1995). Thus, reciprocal rather than unilateral relationships and dyads rather than friendship networks may create specific contexts that are most conducive to socialization of internalizing symptoms. Specifically, due to the cohesiveness and intimacy between member dyads, best friendship relations may be most influential.

## Gender Differences

Peer and friendship relationships of male and female adolescents are substantially different with respect to several domains (for a review, see Rose and Rudolph 2006). Gender differences, such as those pertaining to the structure and content of adolescents’ relationships, may play a central role in influencing peer socialization of depressive symptoms, but not alcohol misuse. This hypothesis relies on the fact that the features of female friendships may facilitate the development of some specific processes underlying peer influence of depressive symptoms, posing female adolescents particularly at risk for socializing their depressive symptoms.

With regard to the social structure of the relationships, female adolescents tend to prefer and interact more frequently in dyadic relationships, whereas male adolescents engage more often in relationships within larger peer groups (e.g., Benenson 1990; Markovits et al. 2001). Concerning the content of the relationships, female adolescents report more intimacy, loyalty and self-disclosure with their closest best friends than male adolescents (e.g., Camarena et al. 1990; Galambos 2004; McNelles and Connolly 1999). Thus, female adolescents may be more likely to share their negative feelings and stressful experiences within their dyadic best friendships and consequently be also more at risk to engage in those maladaptive processes that potentially underlie depression contagion, such as co-rumination (i.e., excessive discussion of problems within dyadic relationships; Rose 2002). Previous studies have provided evidence in this direction, showing that, as compared to male adolescents, female adolescents report higher levels of co-rumination with their best friends (e.g., Hankin et al. 2010; Rose 2002). Moreover, when dealing with stressful situations, female adolescents rely more on their close friends and seek support within their best friendships more than male adolescents do (e.g., Colarossi and Eccles 2000; De Goede et al. 2009). Consequently female adolescents may also be more vulnerable to develop depressive symptoms in front of interpersonal stressors, such as those arising from having a best friend with high depressive symptoms (Rudolph 2002; Starr and Davila 2008). Thus, peer influence of depressive symptoms may specifically occur within female best friendships.

Importantly, not only gender differences in peer relationships, but also the overall tendency of female adolescents to develop higher levels of depressive symptoms (for a review, see Hankin and Abramson 2001) may further contribute to make them more susceptible to depression socialization as compared to male adolescents. Hence, female vulnerability to depressive symptoms may facilitate socialization processes. The opposite pattern may be true with regard to alcohol misuse, in relation to which, though to a less extent (see Schulte et al. 2009), gender differences have been demonstrated in favor of male adolescents (for a review, see White and Huselid 1997). Yet, although male adolescents may be generally somewhat more predisposed to engage in alcohol misuse than female adolescents, unlike depressive symptoms, gender differences on peer relationships may be less relevant when it comes to socialization of drinking behaviors. Indeed, the processes underlying alcohol socialization may take place across different friendship contexts, regardless of the characteristics of the relationships. Prior work provided evidence supporting the hypothesis that peer influence of alcohol may occur equally among male and female adolescents. First, alcohol use has been shown to associate with social status similarly among male and female adolescents (e.g., Allen et al. 2005; Mayeux et al. 2008), which seems to suggest that drinking behaviors may be equally rewarded among both genders. Thus, both male and female adolescents may be reinforced to emulate their friends’ drinking behaviors. Second, no gender differences have been found in relation to imitation of drinking behaviors, which may represent one of the primary processes of peer influence (Caudill and Kong 2001; Larsen et al. 2009, 2010). Though a few exceptions must be acknowledged (e.g., Suls and Green 2003), most previous studies confirmed the absence of gender differences in relation to socialization of alcohol use (e.g., Burk et al. 2012; Kiuru et al. 2010; Rabaglietti et al. 2011).

## The Present Study

Driven by the aforementioned theoretical models, this study aimed to expand the existing literature on peer influence and friendship contexts. To do so, this study employed a longitudinal design (two time points 6 months apart) to examine peer socialization related to adolescent alcohol misuse and depressive symptoms within two levels of friendship, that is, in multiple overlapping relationships within friendship networks and non-overlapping friendship dyads, distinguishing between unilateral and reciprocal relationships. The complementary use of social network and dyadic approaches has several advantages. First, stochastic actor-based models (Snijders et al. 2010) allowed us to examine socialization effects within a friendship network while controlling for effects of selection and network structure. Second, although dyadic analyses have typically been used to examine best friendships, they also may provide more fine-grained and detailed estimates of dyadic similarity, showing in which type of relationships socialization effects are more likely to take place. In this investigation, we collapsed all the friendship ties present in the social network in friendship dyads in order to examine socialization effects within different friendships. Here, based on previous studies, socialization effects were estimated within friendship dyads that were stable across the six months between Time 1 and Time 2 (e.g., Giletta et al. 2011; Laursen et al. 2011; Popp et al. 2008). Specifically, a group of stable reciprocal best friend dyads was identified with the aim to examine the closest and most intimate friendships. In the light of the gender-specific relational patterns that characterize adolescent friendships (Rose and Rudolph 2006), particular attention was given to gender differences when testing these effects.

We hypothesized that peer influence would occur differently across friendship contexts as a function of the specific behavior (i.e., alcohol misuse vs. depressive symptoms). Specifically, based on peer influence theories (e.g., social learning theories; Bandura 1977), we expected socialization of alcohol misuse to occur within best friendships as well as overall within the larger friendship network. On the contrary, in line with interpersonal theories of depression (Coyne 1976), we hypothesized that adolescents’ peer influence concerning depressive symptoms would not take place within the friendship network. Based on these theories and previous social network studies on alcohol consumption and depressive symptoms (e.g., Kiuru et al. 2010; Mercer and Derosier 2010; Rabaglietti et al. 2011), we did not expect socialization effects to differ as a function of gender or friendship type (unilateral and reciprocal friendships) in the network analyses. With respect to the dyadic analyses, we hypothesized socialization effects of alcohol misuse across different stable dyadic relationships, both unilateral and reciprocal. In contrast, we expected socialization effects of depressive symptoms to characterize exclusively reciprocated best friend relationships and to be stronger for female adolescents compared to male adolescents.

## Method

### Participants

Participants were 704 adolescents (53 % male) between 14 and 18 years old (*M* = 15.53 years, *SD* = 1.01) living in a suburban area in northwestern Italy. Adolescents were recruited from 51 classrooms from three public high schools; specifically, 38.1 % of them were enrolled in the first grade, 32.8 % in the second grade, and 29.1 % in the third grade (i.e., from Grade 9 to 11 in the U.S.). Adolescents followed one of three main educational tracks in the Italian secondary educational system (ISTAT 2009), with 37.2 % of them attending pre-university education (38.6 % of the national population), 50.3 % a technical education (38.2 % of the national population) and 12.5 % a vocational education (23.2 % of the national population). At baseline, 85.3 % of the participants lived in an intact family with both biological parents, 12.2 % lived in a single-parent family, and 2.5 % lived in a stepfamily or with other significant relatives. Parental divorce rate was 5.5 % (12 % of the national population) and unemployment rate was 2.2 % (7.8 % of the national population). Parents’ educational level was highly comparable to that of the national population aged 25 to 64 years old (OECD 2009), with 35 % of parents having a level of education lower than high school, 51 % a high school degree, and approximately 14 % a university or post-university degree. Most participants were born in Italy (94.1 %) and had at least one parent who was born in Italy (93.7 %). About 7.6 % of adolescents were ethnic minorities, representing the two main ethnic minority groups residing in Italy: 2.6 % from South-Eastern Europe (e.g., Romania and Albania) and 2.6 % from Morocco (the remaining 2.5 % of participants belonged to other ethnic groups).

### Measures

#### Alcohol Misuse

Alcohol misuse during the past 6 months was assessed by two items adapted from the Youth Risk Behavior Survey (YRBS; Brener et al. 1995). One item measured the frequency of binge drinking (“How often did you have five or more alcoholic drinks in a single occasion within few hours?”) and the second item measured the frequency of drinking to intoxication (“How many times did you get sick or hangover after drinking alcohol?”). Each item was rated on a 7-point Likert scale: 0 = *never*, 1 = *1*–*2 times*, 2 = *3*–*5 times*, 3 = *6*–*9 times*, 4 = *10*–*19 times*, 5 = *20*–*29 times*, 6 = *30 or* *more times*. The frequencies of binge drinking and alcohol intoxication emerged to be highly comparable to those reported in the 2007 ESPAD report (European School Survey Project on Alcohol and Other Drugs), in which two items similar to those employed in the present study have been administered to a large normative sample of Italian adolescents of similar age (see Hibell et al. 2009). The two items were moderately correlated at both time points (*r* = 0.61 and 0.50 at Time 1 and Time 2, respectively); therefore, they were summed to form a composite measure of alcohol misuse in the last 6 months ranging from 0 (*no alcohol* *misuse*) to 12 (*very frequent* *alcohol misuse*) (Cooper 1994). Because stochastic actor-based models requires the behavioral outcomes to be ordinal discrete variables (Snijders et al. 2010), for the social network analyses, the sum scores of alcohol misuse were collapsed into six ordinal categories which reflected the original distribution of alcohol misuse within the sample. The proportion of male and female adolescents in each alcohol category at Time 1 and 2 is reported in Table 1. For the dyadic analyses, a logarithmic transformation was applied to the sum score of alcohol misuse to correct for positive skewness.

**Table 1:** Descriptive statistics of alcohol misuse and depressive symptoms by gender

	Time 1		Time 2	
	Males	Females	Males	Females
Alcohol misuse (*SD*)	1.37 (2.36) ^a^	0.93 (1.75) ^b^	1.83 (2.48) ^c^	1.03 (1.66) ^b^
None (sum score = 0)	57.4 %	60.2 %	44.7 %	56.7 %
Very infrequent (1–2)	22.6 %	28.2 %	27.8 %	28.5 %
Infrequent (3–4)	9.7 %	6.4 %	13.3 %	10.0 %
Some (5–6)	5.2 %	3.4 %	8.6 %	3.3 %
Frequent (7–8)	2.7 %	0.6 %	3.2 %	1.2 %
Very frequent (sum score ≥ 9)	2.4 %	1.2 %	2.4 %	0.3 %
Depressive symptoms (*SD*)	5.61 (4.52) ^a^	7.91 (5.04) ^b^	5.24 (4.46) ^a^	8.26 (4.85) ^b^
None (<−1 *SD*)	15.9 %	4.8 %	23.2 %	4.6 %
Very infrequent	25.0 %	16.0 %	18.6 %	11.6 %
Infrequent	25.3 %	25.7 %	26.5 %	27.1 %
Some	10.2 %	14.2 %	12.7 %	15.2 %
Frequent	10.5 %	17.2 %	9.5 %	16.5 %
Very frequent (≥1 *SD*)	13.1 %	22.1 %	9.5 %	25.0 %

Alcohol misuse scores range between 0 and 12 and depressive symptoms scores between 0 and 26

Raw score of alcohol misuse are presented here however a logarithmic transformation was used for descriptive and dyadic analyses to correct for positive skewness

^a, b, c^ Different subscriptions indicate significant differences

#### Depressive Symptoms

Adolescent depressive symptoms were assessed using the Short Mood and Feeling Questionnaire (SMFQ; Angold et al. 1995). Participants were instructed to rate on a 3-point scale (0 = *not true*, 2 = *true*) 13 items (e.g., “I felt miserable or unhappy”, “I did everything wrong”) describing depressive symptoms during the past 2 weeks. The psychometric properties of this measure have been shown to be satisfactory (Messer et al. 1995) and it has been widely used in studies with adolescent samples (Rothon et al. 2009; Stansfeld et al. 2004). A sum score was computed across all items, with higher values indicating higher depressive symptoms (Cronbach’s *α* = 0.84 at both time points). The mean levels of depressive symptoms within our sample were comparable to those found in previous studies using the SMFQ among normative samples of adolescents of similar age (e.g., Eley et al. 2004; McKenzie et al. 2011). Depressive symptoms were normally distributed, so the sum score of depressive symptoms was used for the dyadic analyses. Moreover, to meet the requirement for stochastic actor-based modeling six ordinal categories were created using a one-half standard deviation from the sample mean score as cut-off point. Participants with a sum score one standard deviation below the sample mean were included in the lowest category (i.e., no depressive symptoms), participants with a sum score between one and one-half standard deviation below the sample mean were included in the “very infrequent” category, those with a sum score between one-half standard deviation below and the sample mean in the “infrequent category” and so on (see Table 1). The sample mean and standard deviation of depressive symptoms computed across the two time points were used to create the categories, in order to ensure that adolescent changes over time in the behavioral categories reflected actual individual changes rather than changes due to distribution differences across time points (see also Rabaglietti et al. 2011). Similar categorization procedures are commonly employed when analyzing social network data with stochastic actor-based models (e.g., Mercer and Derosier 2010; Rabaglietti et al. 2011). The proportion of male and female adolescents in each depression category at Time 1 and 2 is reported in Table 1.

#### Friendship Nominations

Adolescents were provided with a roster of all their grademates from which they were asked to select an unlimited number of friends (“Who are your best, closest friends?”). Within each roster students’ names were presented divided by classroom and alphabetized within each classroom. Each student’s name was associated with a code number and, to ensure participants’ anonymity, adolescents were asked to report on the questionnaire the numbers instead of the names of their best friends. Adolescents were allowed to nominate same- and cross-gender peers and were instructed to rank them in order of importance (i.e., starting with their very best friend, followed by their second best friend, etc.) (Parker and Asher 1993). On average, adolescents selected their friends from a pool of approximately 170 students and did not report any particular difficulty with identifying their friends from the rosters. Similar grade-wise peer nomination procedures have been largely used in previous studies and have been shown to be valid tools to assess adolescent friends and acquaintances (see Poulin and Dishion 2008).

For the social network analyses, an adjacency matrix was created at each time point by including all adolescent friendship nominations. These matrices consisted of 704 rows (nominators) and 704 columns (nominees), with the absence or presence of a friendship tie between a nominator and a nominee indicated by a zero or one, respectively. Because adolescents in different grades could not nominate each other, structural zeros were included in cells between participants in the different reference groups. Such a procedure allowed merging and simultaneously analyzing the different friend networks generated by adolescents attending different grades (Ripley et al. 2011).

For dyadic analyses, each friendship tie was classified into groups based on: (a) the gender of the adolescents involved in the relationship (i.e., male, female and cross-gender dyads), (b) the type of the friendship (i.e., unilateral, reciprocal or mixed across the two time points), and (c) the time of the relationship (i.e., Time 1 only, Time 2 only, or Time 1 and 2). Specifically, we identified the very best friend dyads that were stable across the two time points.

#### Socio-Demographic Variables

Information on adolescent socio-demographic characteristics, including gender, age, and parents’ educational level, was collected through self-reports. With regard to mother and father’s educational level, original response options were collapsed into three categories corresponding to a low (i.e., less than high school), medium (i.e., high school), and high (i.e., post high school or university degree) level of education. The highest educational level of the parents (or the one available in case of missing information) was used as a proxy for SES.

### Procedure

Data for this study were collected as part of a longitudinal project on adolescent peer relationships and internalizing symptoms among a community sample from the Northwest of Italy. This project was approved by the internal ethics committee of the University of Torino. A total of 1,038 families of adolescents attending three high schools were contacted to participate in the study. A letter describing the study was sent to adolescents’ families. The letter asked parents to provide permission for their children to participate in the study. Only 48 (4.6 %) families denied permission and all adolescents whose parents granted consent to participate also provided assent. Adolescents did not receive any incentive for their participation. During school hours, trained research assistants administered identical questionnaires in the fall (Time 1) and 6 months later in the spring of the school year (Time 2). All instruments were administered in Italian and a forward-backward translation procedure was used to translate the measures that were originally developed in English. These measures were examined by bilingual translators and problematic items were discussed to achieve consensus for the final translation.

In order to properly assess socialization effects in both friendship networks and dyads, only participants who were present at both waves of data collection were selected for the analyses. At Time 1, 137 adolescents were absent on the day of the assessment and data from 21 participants were excluded due to unreliable answers (*n* = 6) or disorder diagnosis (e.g., autistic disorders or intellectual disabilities; *n* = 15). The decision to exclude this latter group of participants from the analyses arose from the fact that this study aimed to examine peer influence among typically developing adolescents, whereas peer socialization processes may be expected to be remarkably different for adolescents with similar disorders, due to the well-documented impairment in their interpersonal and social functioning (American Psychiatric Association 2000). Of the 832 adolescents present at Time 1, 711 completed the survey at Time 2. A logistic regression analyses was conducted to test whether adolescents in the longitudinal sample differed in socio-demographic characteristics (i.e., gender, age, ethnicity, educational track, family structure and parents’ educational level), number of friendship nominations, baseline levels of alcohol misuse and depressive symptoms from adolescents who dropped out. Significant differences emerged only in relation to age, indicating that adolescents in the longitudinal sample were younger (*M* = 15.55; *SD* = 1.07) compared to adolescents who were lost to attrition (*M* = 16.06; *SD* = 1.44) (*OR* = 0.70, *p* < 0.001, 95 % CI [0.60, 0.84]). An additional seven participants were excluded from this final sample because they were younger than 14 years or older than 18 years. Thus, the analytic sample included 704 adolescents (67.8 % of the target population).

### Strategy of Analyses

First, stochastic actor-based models of network-behavioral dynamics were conducted using the Simulation Investigation for Empirical Network Analysis software (SIENA; Ripley et al. 2011) to assess peer influence effects within friendship networks without distinguishing between adolescents’ very best friends (i.e., the first friend nominated by each adolescent) and other friends. Stochastic actor-based models are models that allow investigating the co-evolution of social networks and individual behaviors over time. The main feature of this method is that changes over time in individual behaviors and social network are recognized to be strongly interdependent. That is, a change in the behavior of an actor (i.e., an individual in the network) may occur as a consequence of his/her ties with other actors in the network. Similarly, a change in a network tie may be attributable to certain actors’ characteristics. In these models a network tie between two actors is operationalized as a binary variable (i.e., presence or absence of a tie) and actors are assumed to control and voluntarily decide if and when change their outgoing ties with other actors (i.e., form a new tie or dissolve an existing one) as well as their behavior (i.e., increase or decrease their behavior). These methods utilize a continuous time modeling approach, which identifies the most likely sequence of individual changes from the total amount of changes in network ties and individual behaviors between two discrete time points. This allows disentangling selection and socialization controlling for alternative (unobserved) processes that may take place in-between two observed measurements and that, if neglected, may lead to erroneous conclusions about the mechanisms underlying the observed changes (see Steglich et al. 2010). While the complexity of these models does not allow for the explicit calculation of effects, parameter values and their standard errors can be estimated using an iterative simulation procedure. For each parameter estimated, a *t*-value can be calculated by dividing the parameter estimate by its standard error. Moreover, a *t*-ratio for convergence is also generated for each parameter in the model, indicating the discrepancies between the simulated values and the observed ones. T-ratios lower than 0.1 in absolute value indicate good algorithm convergence. Additional information about stochastic actor-based models as well as the mathematical formulae for each parameter can be found elsewhere (Snijders et al. 2007, 2010; Steglich et al. 2010; Veenstra and Steglich 2012).

Two stochastic actor-based models were tested. The first examined socialization effects of alcohol misuse and depressive symptoms, and the second model also included interactions testing moderation effects of gender and friendship reciprocity on peer socialization. Three dependent variables were simultaneously modeled within each model. Two represented behavioral dynamics (i.e., describing changes in alcohol misuse and depressive symptoms over time) and one represented network dynamics (i.e., describing changes in friendship ties over time). Concerning behavioral dynamics, changes in adolescent alcohol misuse and depressive symptoms were predicted by friends’ behaviors (i.e., socialization effects). Specifically, socialization effects were operationalized in terms of the tendency of adolescents to become more similar to the behaviors of their peers with whom they had a friendship tie at Time 1, accounting for the number of adolescent outgoing ties (i.e., total similarity; see also Snijders et al. 2010). For both alcohol misuse and depressive symptoms, control parameters included individual differences in behaviors (i.e., linear and quadratic shape) and the main effects of adolescent socio-demographic characteristics (i.e., gender, age, educational track, and parent educational level). Moreover, socialization effects were estimated while accounting for network dynamics. Friendship formation was predicted by two different sets of parameters, corresponding to network structural effects (i.e., reciprocity and network closure effects) and effects related to characteristics of ego (i.e., nominator) and alter (i.e., nominee), such as behavioral similarity between ego and alter (i.e., friendship selection). Thus, socialization effects were estimated while accounting for network structural and selection effects.

Second, dyadic correlations were used to investigate peer influence in different types of friendships. Socialization by peers at the dyadic level was assessed by examining increases in similarities in alcohol misuse and depressive symptoms between dyad members over time. To do so, intraclass correlations (ICCs) representing the degree of behavioral similarity between dyad members were calculated for stable friend dyads at both time points. Differences in the magnitude of the ICCs at Time 1 and Time 2 were tested using correlational contrasts designed to examine correlated but non-overlapping correlations with a *Z* Pearson-Filon statistic (*ZPF*; Raghunathan et al. 1996). To deal with the random assignment of some adolescents as targets and others as friends (i.e., the indistinguishable nature of the dyad partners), we utilized the pairwise approach recommended by Griffin and Gonzalez (1995). This requires all relationships to be entered twice, once with the target adolescent’s scores entered first and the friend’s score entered second, and once with the friend’s scores entered first and the target adolescent’s scores entered second. The statistical significance of the pairwise correlations and the correlational contrasts is based on the “effective sample size”, which falls between the actual sample size (i.e., number of dyads times two) and the number of dyads depending on the magnitude of similarity (see p. 432 Griffin and Gonzalez 1995).

## Results

### Descriptive Analyses

Table 1 presents descriptive statistics of alcohol misuse and depressive symptoms by gender across time points. A 2 (time) x 2 (gender) repeated measures MANOVA was performed to test gender differences on mean levels of alcohol misuse and depressive symptoms at Time 1 and 2. Significant main effects of time, *F*(2, 686) = 15.99, *p* < 0.001, and gender, *F*(2, 686) = 45.90, *p* < 0.001, were qualified by a two-way interaction, *F*(2, 686) = 7.14, *p* = 0.001. The interaction effect for alcohol misuse was significant, *F*(1, 687) = 8.40, *p* = 0.004. Follow-up analyses showed that alcohol misuse tended to significantly increase over time for male adolescents, *F*(1, 368) = 36.01, *p* < 0.001, but not for female adolescents, *F*(1, 324) = 3.45, *p* = 0.064. For depressive symptoms, only the main effect of gender emerged as statistically significant, *F*(1, 687) = 69.67, *p* < 0.001, indicating that female adolescents reported higher levels of depressive symptoms compared to male adolescents at both time points *F*(1, 687) = 69.67, *p* < 0.001.

### Stochastic Actor-Based Models

The indices of network structure indicated that overall friend networks tended to become more cohesive over time. Specifically, at Time 2, an increase in the average number of outgoing nominations (i.e., average degree; from 5.47 to 5.98) generated a growth of approximately 10 % in the total number of friendship ties (from 3,850 to 4,210). Time differences were noted also with regard to the reciprocity index (0.52 and 0.55 at Time 1 and Time 2, respectively) and transitivity index (0.32 and 0.37 at Time 1 and Time 2, respectively), suggesting that the number of reciprocal nominations as well as triadic relations demonstrating transitive network closure (i.e., my friends are also friends) tended to slightly increase from Time 1 to Time 2. Overall, between the two time points a satisfactory number of changes in adolescent behaviors (39.5 % of adolescents changed their alcohol behavior and 67.9 % their depressive symptoms over time) and friendship ties (indicated by a Hamming distance of 3,380 and a Jaccard index equal to 0.41) was observed. This suggests an adequate amount of changes in friendships and individual behaviors between baseline and follow-up to estimate socialization as well as selection effects (see for further clarifications: Veenstra and Steglich 2012).

#### Behavioral Dynamics: Socialization Effects

Socialization effects on alcohol misuse and depressive symptoms were initially estimated while controlling for several socio-demographic characteristics, including gender, age, educational track, and parent educational level. However, because only gender and age emerged as significant predictors (gender for both alcohol misuse and depressive symptoms, age for alcohol misuse only; see Appendix), a more parsimonious model was estimated that excluded non-significant parameters (following Snijders et al. 2010). The final complete model including all the parameter estimates is reported in the Appendix. Algorithm convergence emerged to be excellent, with *t*-ratios lower than 0.1 for all parameter estimates.

Concerning alcohol misuse, after controlling for the effects of individual tendencies (i.e., linear and quadratic shape), gender, and age, a positive significant effect was found for the total similarity parameter. This parameter showed evidence for peer socialization, indicating that adolescents’ alcohol behavior tended to become more similar to their friends’ alcohol behavior over time (see Table 2). However, with regard to depressive symptoms, after controlling for individual differences and adolescent gender, the total similarity parameter did not emerge as significant, implying absence of socialization effects within the social network (see Table 2). In the second model, four interactions testing whether gender and reciprocity moderated peer socialization of alcohol misuse and depressive symptoms emerged as nonsignificant, suggesting that peer socialization of both behaviors did not differ as a function of adolescent gender or friendship reciprocity.

**Table 2:** Stochastic actor-based model parameter estimates for socialization and selection effects on alcohol misuse and depressive symptoms

Parameters	Estimate	S.E.	*p*-value
Socialization effects			
Alcohol total similarity	0.48	0.15	0.001
Depression total similarity	0.04	0.07	0.569
Selection effects			
Alcohol ego	0.11	0.02	<0.001
Alcohol alter	0.11	0.02	<0.001
Alcohol similarity (homophilic selection)	0.83	0.17	<0.001
Depression ego	−0.02	0.01	0.136
Depression alter	0.00	0.01	0.908
Depression similarity (homophilic selection)	0.39	0.13	0.002

The complete estimated model is reported in Appendix

#### Network Dynamics: Selection Effects

Although not the primary focus of this investigation, we briefly describe other parameters specified in the actor-based models, including effects of homophilic selection (see Appendix for the complete model). All network structural effects emerged as significant, indicating that adolescents were selective in choosing their friends (negative outdegree), were likely to reciprocate their friendship ties (positive reciprocity), and were likely to form cohesive triadic relationships demonstrating local hierarchies (positive transitivity triplets and negative three-cycle effect). With respect to effects related to the individual characteristics of the nominators and nominees, the results suggested that friendship formations were more likely to occur between adolescents who attended the same classroom (positive same class) and between same-gender adolescents (positive gender similarity). In addition, significant effects emerged for ego (i.e., nominator) and alter (i.e., nominee) parameters of alcohol misuse, indicating that adolescents with high levels of alcohol misuse tended to nominate more friends (outgoing nominations: alcohol ego) and to receive more nominations from other peers in the network (ingoing nominations: alcohol alter; network-popularity) compared to adolescents reporting low alcohol misuse (see Table 2). No significant differences emerged between adolescents reporting different levels of depressive symptoms with respect to their network activity (outgoing nominations: depression ego) and network-popularity (ingoing nominations: depression alter). Finally, significant positive similarity effects were found for both alcohol misuse and depressive symptoms (see Table 2). These parameters showed evidence for homophilic selection effects, indicating that adolescents tended to form friendships with peers similar to themselves in terms of alcohol misuse and depressive symptoms.

### Dyadic Analyses

Overall, 70 % of the dyadic friendships were same-gender (41 % male dyads and 29 % female dyads) and 30 % were cross-gender. Concerning the type of dyad, 61.4 % of the relationships were unilateral (i.e., unilateral tie at one of the two time points only and no tie at the other, or unilateral tie at both time points), 24.5 % were reciprocal (i.e., reciprocal tie at one of the two time points only or at both time points), and 14.1 % were mixed (i.e., unilateral tie at one of the two time points and reciprocal at the other time point). Differences existed in the type of relationship between same- and cross-gender dyads, with the former being overrepresented in the reciprocal (27.6 % vs. 17.2 %) and mixed (16.1 % vs. 9.5 %) types and the latter in the unilateral type (73.3 % vs. 56.3 %), *χ* ^2^(2) = 104.77, *p* < 0.001. Male and female dyads also differed from each other, with male adolescents being more likely to have unilateral relationships (60.3 % vs. 50.5 %) and female adolescents reciprocal relationships (33.7 % vs. 23.4 %), *χ* ^2^(2) = 38.17, *p* < 0.001. With regard to very best friend dyads that were stable across the two time points, only one was cross-gender. Moreover, female adolescents reported a higher percentage of very best friendships stable over time than male adolescents (3.3 % vs. 1.7 %), *χ* ^2^(1) = 7.12, *p* = 0.008.

#### Socialization Effects

Socialization effects could be identified within friendships that were stable over time, here defined as those relationships in which at least one unilateral tie from the same dyad member (i.e., outgoing tie) was present at both time points. In these friendships, evidence for socialization effects may be shown if similarities between dyad members at Time 2 emerged to be significantly higher than similarities at Time 1. Such a procedure allows for the identification of acquired similarity that may be attributed to peer socialization while adjusting for pre-existing similarity (i.e., homophilic selection). For stable friendships, intraclass correlations (ICCs) between dyad members’ alcohol misuse and depressive symptoms were computed separately for each relationship type at both time points. Additional partial correlations controlling for the number of times each adolescent’s score appeared within each dyadic relationship were also conducted in order to account for unequal individual contributions. Because partial correlations were highly similar to ICCs, with identical significance levels, ICCs are presented in Table 3 to allow for easier interpretation.

**Table 3:** Intraclass correlations at Time 1 and Time 2 for examining socialization effects on alcohol misuse and depressive symptoms by dyad type and gender

	Time 1			Time 2		
	Male	Female	Cross-gender	Male	Female	Cross-gender
Alcohol misuse						
Unilateral	0.20***	0.24**	0.06	0.22***	0.26**	0.14
Reciprocal	0.36***	0.12	0.29*	0.36***	0.36***	0.16
Reciprocal very best	0.70**	0.29	–	0.47*	0.46**	–
Unilateral T1 reciprocal T2	0.26**	−0.03	0.15	0.32***	0.29**	0.20
Reciprocal T1 unilateral T2	0.12	0.32**	−0.03	0.07	−0.03	−0.04
Depressive symptoms						
Unilateral	0.23***	−0.01	0.05	0.04	0.14	−0.06
Reciprocal	0.23***	−0.03	0.06	0.15*	0.04	−0.07
Reciprocal very best	−0.05	0.07	–	-0.07	0.35*	–
Unilateral T1 reciprocal T2	0.00	0.15	0.07	0.08	−0.13	−0.19
Reciprocal T1 unilateral T2	−0.01	0.12	0.07	−0.09	0.04	0.02

Significance levels are adjusted according to Griffin and Gonzalez 1995. * *p* < 0.05. ** *p* < 0.01. *** *p* < 0.001

Regarding alcohol misuse, at Time 2 significant ICCs were found across different types of friendships for both male and female same-gender dyads but not for cross-gender dyads, with effects ranging from small to medium-large (*r* ^*2*^ range 0.22–0.47). These indicated concurrent similarity in alcohol misuse across all relationship types. For these eight dyad groups with significant ICCs at Time 2, correlation contrasts were used to test differences between Time 1 and Time 2. Only three emerged to be statistically significant. Similarity in alcohol misuse at Time 2 was higher than at Time 1 for female adolescents in reciprocal dyads and in dyads that were unilateral at Time 1 and reciprocal at Time 2 (*ZPF* = 4.33; *p* < 0.001 and *ZPF* = 3.70; *p* < 0.001, respectively). On the contrary, similarity at Time 2 was actually lower than at Time 1 for male adolescents in reciprocal very best friendships (*ZPF* = 2.04; *p* = 0.041). Concerning depressive symptoms, similarities at Time 2 were statistically significant for male adolescents in reciprocal dyads and female adolescents in reciprocal very best friendship dyads (*r* ^*2*^ 0.15 and 35, respectively). Only one of the two correlational contrasts revealed a significant difference in similarity at Time 1 and Time 2; similarity in depressive symptoms at Time 2 was higher than at Time 1 for female adolescents within reciprocal very best friendships (*ZPF* = 1.97; *p* = 0.049). Importantly, levels of depressive symptoms did not differ between female adolescents involved in very best friendship dyads and other female adolescents in the sample at Time 1 (*M* = 8.06 vs. 7.46) or Time 2 (*M* = 8.32vs. 8.27). Overall, these results indicate that across the two time points, female dyad members became more similar to each other in their levels of alcohol misuse (in reciprocal dyads and in dyads that were unilateral at Time 1 and reciprocal at Time 2) and depressive symptoms (in very best friendships), providing evidence for socialization effects.

## Discussion

Substantial research has examined peer socialization effects with regard to a wide variety of possible attitudes, cognitions, and behaviors (for a review, see Prinstein and Dodge 2008). However, relatively little attention has been dedicated to the *types* of peer relationships that may be particularly potent for peer socialization to occur and to whether these different friendship contexts may yield differential risk for peer socialization across different behaviors. The present study addressed this long-standing gap in the literature by simultaneously examining adolescent peer influence of alcohol misuse and depressive symptoms in diverse friendship contexts while differentiating between the relationship level (i.e., best friendships and friendship networks) and reciprocity (unilateral and reciprocal). In line with our predictions, the results showed that peer influence differed across the contexts of friendships as a function of the specific behavioral domain. In friendship networks, after controlling for selection effects, peer socialization emerged for alcohol misuse but not for depressive symptoms regardless of the reciprocity of the relationships. Conversely, within friendship dyads, evidence for peer socialization of both alcohol misuse (in reciprocal dyads and in dyads that were unilateral at Time 1 and reciprocal at Time 2) and depressive symptoms (exclusively in very best friendships) was found for female adolescents only. This research adds to the current literature and advances our knowledge of adolescent peer influence by highlighting the domain specificity (e.g. substance use versus internalizing problems/depressive symptoms) of peer socialization, and the crucial role of the friendship context.

In line with social learning theories (e.g., Bandura 1977), our findings indicate that adolescents conformed to the drinking behaviors of their friends within broader friendship networks. Here, socialization of behaviors may occur through processes of imitation and modeling (e.g., Larsen et al. 2009, 2010) as well as active persuasion, which may easily take place within large peer groups (e.g., Bot et al. 2007; Overbeek et al. 2011). Due to the visibility of alcohol consumption and its’ externalized social nature, within a network context adolescents may be inclined to engage in alcohol misuse simply because they observe their friends doing it. Moreover, reinforcement mechanisms (both vicarious and direct) and positive expectancies associated with alcohol misuse, such as sociability and achievement of status among peers, may exacerbate these modeling processes (e.g., Allen et al. 2005; Bot et al. 2005a). Although in our study we did not directly assess these mechanisms, the additional finding that alcohol misuse was associated with a higher number of received nominations within friendship networks seems to further support these theoretical models (e.g., social learning theories). That is, increasing friendship nominations (i.e., network centrality) within peer groups may not only further reinforce high levels of alcohol consumption (e.g., Mayeux et al. 2008) but also peer socialization of alcohol. Indeed, as it has been shown in prior work, high status adolescents, such as adolescents who hold a central position in their social networks, are particularly influential and likely to be emulated by other peers (e.g., Cohen and Prinstein 2006). Thus, it may be assumed that peer group norms as well as group dynamics play a central role in contributing to the spread of alcohol consumption within large peer groups.

This theoretical framework may also explain the findings showing that socialization effects occurred within friendship networks both in reciprocal as well as unilateral relationships. That is, although socialization in reciprocal friendships may involve imitation processes as well as interpersonal dynamics that include more direct forms of reinforcement (e.g., peer pressure, deviancy training; Dishion and Owen 2002; Graham et al. 1991), imitation and modeling processes may take place in less intimate relationships due to the social nature and the motives associated with alcohol misuse (e.g., in particular social and enhancements motives; for a review, see Kuntsche et al. 2005). Therefore, within friendship networks, reciprocal as well as unilateral relationships may be relevant in affecting adolescents’ drinking behavior. Overall, our results showing socialization effects, as well as homophilic selection, of alcohol misuse in friendship networks are consistent with the existing peer influence literature (e.g., Burk et al. 2012; Kiuru et al. 2010; Knecht et al. 2011).

In contrast, socialization of depressive symptoms was revealed only within dyadic friendships, and exclusively between female adolescents in reciprocated very best friendships. This suggests that this type of peer socialization may involve different mechanisms than those responsible for alcohol socialization. In other words, it is perhaps unlikely that depressive symptom socialization occurs via modeling and imitation processes. Instead, according to interpersonal theories of depression (Coyne 1976; Joiner and Timmons 2009), contagion may occur when adolescents with high levels of depressive symptoms engage in maladaptive interpersonal interactions, breeding negative emotional states in their relational partners and possibly exacerbating their depressive symptoms. These processes take place when people directly interact with and disclose to each other within dyadic relationships. That is, relationships with high levels of closeness and intimacy between partners, which may exist in reciprocated best friendships (e.g., Buhrmester 1990), may be specifically vulnerable to socialization of emotional states (e.g., Van Orden and Joiner 2006). The fact that peer influence of depressive symptoms only emerged in female best friend dyads is also consistent with the hypotheses regarding depression related social behaviors (e.g., co-rumination, excessive reassurance seeking) as potential underlying mechanisms of depression socialization. Co-rumination, defined as an excessive discussing of personal problems (Rose 2002; Rose et al. 2007), has been described as a dyadic phenomenon which specifically involves members of close relationships, and it has been found to predict increases in depressive symptoms over time, especially for female adolescents (Hankin et al. 2010; Rose et al. 2007). Thus, considering that female adolescents, as compared to male adolescents, engage in more intimate and close relationships and tend to report higher levels of self-disclosure (e.g. Buhrmester and Furman 1987; Parker and Asher 1993; Sharabany et al. 1981) as well as co-rumination (e.g., Hankin et al. 2010; Rose 2002; Rose et al. 2007), it is not surprising that they are specifically at risk for depression socialization. This finding is consistent with previous studies on depression socialization in adolescent dyadic friendships (e.g., Stevens and Prinstein 2005) and replicates a study conducted with a sample of adolescents from a different population (Giletta et al. 2011), in which female but not male adolescents were shown to be affected by their best friends’ depressive symptoms over time.

However, these results are not in line with other recent studies that, using a similar methodology (i.e., stochastic actor-based models), found evidence for peer socialization of depressive symptoms within friendship networks of children (Mercer and Derosier 2010) and adolescents (Kiuru et al. 2011; Van Zalk et al. 2010). Regarding the study by Mercer and Derosier (2010), differences in the sample age, middle childhood as compared to adolescence in our study, might explain the discordant results. During the transition from childhood to adolescence, dyadic relationships increase in importance and become the main structure of interpersonal interactions, especially for female adolescents (Rose and Rudolph 2006). Thus, adolescent intimacy and self-disclosure with best friends represent common interpersonal processes; conversely, these processes may indistinctly take place with several relational partners during childhood. Concerning the studies by Kiuru and colleagues (2011) and Van Zalk and colleagues (2010), discrepancies in the findings likely result from differences in the study design and model specifications. In the study by Kiuru and colleagues (2011) friendship nominations were restricted to three peers only. Therefore, as compared to our study, socialization effects have been examined within peer networks which included exclusively some friendship relations, likely the closest and more intimate. Moreover, in that study depression socialization was estimated without controlling for socialization effects related to other behaviors, such as alcohol use, which may have partially affected the findings. Finally, in the study by Van Zalk and colleagues (2010), peer socialization effects of depressive symptoms were estimated while accounting for the effects of friends’ drinking and delinquency on adolescents’ depressive symptoms, but not for socialization effects of delinquency and alcohol. Differently, in our study we estimated socialization effects related to depression and alcohol in a multivariate model in which both adolescents’ depressive symptoms and alcohol misuse simultaneously were entered as dependent variables. This allowed for a direct estimation of peer socialization related to one behavior (e.g., depression), while controlling for socialization related to the other behavior (e.g., alcohol misuse).

Interestingly and unexpectedly, whereas female adolescents within dyadic relationships also appeared to be at risk for alcohol socialization, this did not seem to be the case for male adolescents. Here, results emerged to be more complex than expected. Indeed, although we did not anticipate gender differences with regard to socialization of alcohol misuse, neither within friendship networks nor within dyads, results seem to imply that even if both male and female adolescents socialize their drinking behaviors, they may do so in different contexts. These findings are partially in line with prior research suggesting that female adolescents may be more likely to conform to their friend’s behaviors within dyadic and close relationships, and male adolescents in larger peer groups (Berndt and Keefe 1995). Thus, it might be the case that for female adolescents socialization effects of alcohol misuse found in friendship networks mainly reflect peer influence within certain dyadic friendships (i.e., reciprocal dyads and dyads that became reciprocal over time). Conversely, male adolescents may be more susceptible to the influence of their friends within a network context. These differences may be understood in the light of gender differences in peer relationships, indicating that female rather than male adolescents tend to spend more time in dyadic interactions with their friends (for a review, see Rose and Rudolph 2006). Thus, these gender-specific patterns may place female adolescents at a higher risk of peer contagion within dyadic relationships. In line with these models, findings also indicate that the reciprocity of the relationship seems to be an important feature for female adolescents in order to socialize their behaviors. Indeed, no socialization effects were found within unilateral dyads. Conversely, not only male adolescents were not found to socialize their drinking behaviors within dyadic relationships, but also within reciprocal very best friend dyads they emerged to decrease their similarity over time. It might be the case that such unexpected result is partially due to the high initial dyadic similarity in this group of dyads (ICC = 0.70), that likely resulted from both selection and socialization processes. It might be also speculated that for male adolescents relational closeness and intimacy may attenuate peer influence processes. Hence, socialization may be particularly relevant at the beginning of a friendship and may tend to decrease when the friendship is already consolidated and become more intimate and stable over time. Future work assessing friendship duration is needed to shed further light on these gender-specific processes.

Findings from this study have important methodological and theoretical implications for the future examination of peer socialization effects. The results clearly suggest that not all peer influence is the same, but instead, different peer contexts may exert different influences across different behaviors. Thus, future work on peer influence should offer a clear rationale for the specific friendship context under examination, and explain why the relationship context may be relevant especially for the socialization phenomenon of interest. Focusing exclusively on some relationships (e.g., best friendships) while neglecting others may lead to biased and equivocal results. For example, although socialization effects of alcohol misuse were found for male adolescents within social networks, male adolescents did not seem to be affected by their friends’ alcohol consumption in our study at the dyadic level. Thus, collapsing adolescent social networks into dyadic relationships may mitigate apparent influence effects, suggesting that network dynamics matter (Haynie 2001) and that the large peer group is more than simply the sum of its dyadic relationships (Carrington et al. 2005). On the other hand, exclusively relying on social network analyses without examining relational differences may hide the importance of some relationships, as indicated by our finding that depression contagion occurs only in female very best friendships. To date, stochastic actor-based models are not capable of providing more fine-grained results that would differentiate between relationships in which socialization is most likely to occur. Thus, when studying socialization processes within large peer groups, examining moderators of peer influence may be crucial in order to understand under what conditions socialization effects differ. Alternatively, the use of dyadic analyses seems to provide a valuable strategy to complement social network approaches.

Also, this study offers important implications for future research investigating moderators of peer socialization. While prior research has examined moderators of peer socialization indiscriminately across adolescent friendships (Dishion and Tipsord 2011), this study suggests that peer socialization may occur differently across different friendships and behaviors. Thus, moderators of peer socialization effects and measures of peer influence susceptibility may also vary across different forms of influence as well as friendship contexts. In addition to gender, as observed in this study, previously examined moderators may be most relevant for understanding different adolescent susceptibility to dyadic socialization versus larger peer group socialization. For instance, the quality of a friendship may be expected to specifically moderate socialization effects within dyadic relationships rather than friendship networks (e.g., Prinstein 2007). Here, friendship quality may affect peer socialization related to different behavioral domains (e.g., externalizing vs. internalizing behaviors) differently. Conversely, within large peer groups, network characteristics (e.g., network density; Haynie 2001) as well as peer characteristics (e.g., popularity), may exacerbate adolescent contagion, especially in relation to certain behaviors (e.g., externalizing vs. internalizing behaviors). Future research is strongly needed to further test these hypotheses.

### Limitations and Conclusions

In addition to the strengths of this study, including the simultaneous analyses of socialization of two behaviors (alcohol misuse and depressive symptoms) across different contexts of friendship as well as the use of sophisticated data analyses techniques (i.e., stochastic actor-based models), some limitations need to be acknowledged. First, the processes underlying peer socialization have not been examined directly. Therefore, the mechanisms, which contribute to the different forms of influence, remain unknown. Specifically, although based on existing theoretical models of peer influence (e.g., Bandura 1977; Coyne 1976) we assumed that differences in socialization processes may have accounted for different socialization effects across friendship contexts, future research is needed to test this hypothesis. Second, although the longitudinal design allowed identifying socialization effects, the use of only two waves of data collection and the limited age range of the sample (78 % of participants were between 15 and 17 years old at baseline) does not allow drawing conclusions about the developmental course of socialization from childhood to adolescence. Differences across developmental periods exist in relation to the structure of adolescent relationships (e.g., Rubin et al. 1998), the relevance of friendships, as well as adolescent susceptibility to peer influence (e.g., Steinberg and Monahan 2007). These developmental differences may be expected to affect peer influence processes. For instance, dyadic friendships may become at risk for depression contagion particularly with the transition from childhood to adolescence, as evidence showed that self-disclosure, intimacy, and co-rumination tend to increase with age in dyadic relationships, especially for female adolescents (e.g., Buhrmester 1990; Rose 2002).

Third, socialization effects are operationalized in a slightly different manner within the social network and dyadic analyses. Specifically, the continuous-time dynamic nature of the stochastic actor-based models allows for the estimation of unobserved changes between the two observations, as well as the simultaneous estimation of selection and socialization effects. The dyadic analyses, like all other static modeling approaches used to examine peer influence, rely exclusively on observed changes (see Steglich et al. 2010). Thus, results from the two methodologies are not directly comparable, and any differences between these sets of results should be interpreted with caution. Fourth, although similar to the proportion identified in previous work (e.g., Giletta et al. 2011), only a small number of reciprocated very best friend dyads were stable over time in our sample, which may have contributed to the small effect sizes in dyadic correlations. Therefore, future research employing larger samples as well as samples from different populations is warranted in order to generalize the findings. Fourth, analyses were limited to the investigation of peer influence of alcohol misuse and depressive symptoms. Thus, in the light of the behavioral-specificity of socialization processes, these results cannot be generalized to peer influence of other externalizing and internalizing problems. For example, although we might speculate that contagion of other emotional states works similarly to depression contagion, alternative processes cannot be excluded. Finally, this study did not examine socialization effects within peer groups and friendships outside the school context. Yet, it is worth noting that in addition to the analysis of dyadic friendships and multiple friendships within peer networks, the analysis of peer groups, such as friendship cliques, represents a third important friendship context to consider (Poulin and Chan 2010). Evidence suggests that peer socialization of both alcohol and depression also may occur within peer groups (Conway et al. 2011; Urberg et al. 1997); thus, future research is needed to link these findings together in order to identify those peer contexts which uniquely contribute to peer socialization. Moreover, particular attention needs to be given to adolescent friendships outside the school contexts, which may be more intimate and therefore, with regard to some behaviors, more influential (e.g., Kiesner et al. 2003; Van Zalk et al. 2010). For similar reasons romantic relationships would also deserve future consideration because they may be particularly at risk for certain socialization processes (e.g., depression contagion), as it has been shown in the adult literature (e.g., Katz et al. 1999).

Despite these limitations, this study offers important evidence to support a new direction in peer influence research, emphasizing the specific friendships that may yield peer socialization effects of specific behaviors. Future research examining the types of behaviors that may most likely be socialized in different types of peer relationships as well as factors that may differentially affect susceptibility to influence across different contexts and behaviors, will be essential to ultimately mitigate deleterious peer influence effects in youth.

## Appendix

**Table 4:** Parameter estimates for stochastic actor-based model of friendship network, depressive symptoms and alcohol misuse

Parameters	Estimate	S.E.	*p*-value
Network dynamics			
Rate function	11.90	0.34	<0.001
Outdegree	−2.69	0.03	<0.001
Reciprocity	1.57	0.05	<0.001
Transitivity	0.34	0.01	<0.001
3-cycles	−0.37	0.02	<0.001
Same class	1.17	0.03	<0.001
Gender similarity	0.39	0.03	<0.001
Alcohol ego	0.11	0.02	<0.001
Alcohol alter	0.11	0.02	<0.001
Alcohol similarity (homophilic selection)	0.83	0.17	<0.001
Depression ego	−0.02	0.01	0.136
Depression alter	0.00	0.01	0.908
Depression similarity (homophilic selection)	0.39	0.13	0.002
Alcohol dynamics			
Rate function	2.06	0.19	<0.001
Linear shape	−0.37	0.07	<0.001
Quadratic shape	0.10	0.03	0.006
Total similarity (socialization)	0.48	0.15	0.001
Effect from gender	−0.35	0.11	<0.001
Effect from age	0.14	0.06	0.013
Depression dynamics			
Rate function	4.85	0.40	<0.001
Linear shape	−0.01	0.03	0.810
Quadratic shape	0.01	0.02	0.420
Total similarity (socialization)	0.04	0.07	0.566
Effect from gender	0.32	0.06	<0.001

## References

[CR1] Allen JP, Porter MR, McFarland FC, Marsh P, McElhaney KB (2005). The two faces of adolescents’ success with peers: adolescent popularity, social adaptation, and deviant behavior. Child Development.

[CR2] Aloise-Young PA, Graham JW, Hansen WB (1994). Peer influence on smoking initiation during early adolescence: a comparison of group members and group outsiders. Journal of Applied Psychology.

[CR3] American Psychiatric Association (2000). *Diagnostic and**statistical manual**of mental**disorders* (4th ed., text revision). Washington, DC: Author.

[CR4] Angold A, Costello EJ, Messer SC, Pickles A (1995). Development of a short questionnaire for use in epidemiological studies of depression in children and adolescents. International Journal of Methods in Psychiatric Research.

[CR5] Bandura A (1977). Social learning theory.

[CR6] Benenson JF (1990). Gender differences in social networks. Journal of Early Adolescence.

[CR7] Berndt TJ (1982). The features and effects of friendship in early adolescence. Child Development.

[CR8] Berndt TJ, Keefe K (1995). Friend’s influence on adolescents’ adjustment to school. Child Development.

[CR9] Bot SM, Engels RCME, Knibbe (2005). The effect of alcohol expectancies on drinking behaviour in peer groups: observations in a naturalistic setting. Addiction.

[CR10] Bot SM, Engels RCME, Knibbe RA, Meeus WHJ (2005). Friend’s drinking behaviour and adolescent alcohol consumption: the moderating role of friendship characteristics. Addictive Behaviors.

[CR11] Bot SM, Engels RCME, Knibbe RA, Meeus WHJ (2007). Sociometric status and social drinking: observation of modelling and persuasion in young adult peer groups. Journal of Abnormal Child Psychology.

[CR12] Brechwald WA, Prinstein MJ (2011). Beyond homophily: a decade of advances in understanding peer influence processes. Journal of Research on Adolescence.

[CR13] Brener ND, Collins JL, Kann L, Warren CW, Williams BI (1995). Reliability of the youth risk behavior survey questionnaire. American Journal of Epidemiology.

[CR14] Buhrmester D (1990). Intimacy of friendship, interpersonal competence, and adjustment during preadolescence and adolescence. Child Development.

[CR15] Buhrmester D, Furman F (1987). The development of companionship and intimacy. Child Development.

[CR16] Burk WJ, Van der Vorst H, Kerr M, Stattin H (2012). Alcohol intoxication frequency and friendship dynamics: selection and socialization in early, middle and late adolescent peer networks. Journal of Studies on Alcohol and Drugs.

[CR17] Camarena PM, Sarigiani PA, Peterson AC (1990). Gender-specific pathways to intimacy in early adolescence. Journal of Youth and Adolescence.

[CR18] Carrington P, Scott J, Wasserman S (2005). Models and methods in social network analysis.

[CR19] Caudill BD, Kong FH (2001). Social approval and facilitation in predicting modeling effects in alcohol consumption. Journal of Substance Abuse.

[CR20] Cohen GL, Prinstein MJ (2006). Peer contagion of aggression and health risk behavior among adolescent males: an experimental investigation of effects on public conduct and private attitudes. Child Development.

[CR21] Colarossi LG, Eccles JS (2000). A prospective study of adolescents’ peer support: gender differences and the influence of parental relationships. Journal of Youth and Adolescence.

[CR22] Cole DA, Tram JM, Martin JM, Hoffman KB, Ruiz MD, Jacques FM (2002). Individual differences in the emergence of depressive symptoms in children and adolescents: a longitudinal investigation of parents and child reports. Journal of Abnormal Psychology.

[CR23] Conway, C. C., Rancourt, D., Adelman, C. B., Burk, W. J., & Prinstein, M. J. (2011). Depression socialization within friendship groups at the transition to adolescence: the roles of gender and group centrality as moderators of peer influence. *Journal of Abnormal Psychology*. doi:10.1037/a0024779. 10.1037/a0024779 PMC3353414 21842961

[CR24] Cooper ML (1994). Motivations for alcohol use among adolescents: development and validation of a four-factor model. Psychological Assessment.

[CR25] Coyne JC (1976). Toward an interactional description of depression. Psychiatry.

[CR26] De Goede IHA, Branje SJT, Meeus WHJ (2009). Developmental changes and gender differences in adolescents’ perceptions of friendships. Journal of Adolescence.

[CR27] Dishion TJ, Owen lD (2002). A longitudinal analysis of friendships and substance use: bidirectional influence from adolescence to adulthood. Developmental Psychology.

[CR28] Dishion TJ, Tipsord JM (2011). Peer contagion in child and adolescent social and emotional development. Annual Review of Psychology.

[CR29] Dishion TJ, Spracklen KM, Andrews DW, Patterson GR (1996). Deviancy training in male adolescent friendships. Behavior Therapy.

[CR30] Eley TC, Liang H, Plomin R, Sham P, Sterne A, Williamson R, Purcell S (2004). Parental familial vulnerability, family environment, and their interactions as predictors of depressive symptoms in adolescents. Journal of the American Academy of Child and Adolescent Psychiatry.

[CR31] Galambos NL, Lerner RM, Steinberg L (2004). Gender and gender role development in adolescence. Handbook of adolescent psychology.

[CR32] Giletta M, Scholte RHJ, Burk WJ, Engels RCME, Larsen JK, Prinstein MJ, Ciairano S (2011). Similarity in depressive symptoms in adolescents’ friendship dyads: selection or socialization?. Developmental Psychology.

[CR33] Graham JW, Marks G, Hansen WB (1991). Social influence processes affecting adolescent substance use. Journal of Applied Psychology.

[CR34] Griffin D, Gonzalez R (1995). Correlational analysis of dyad-level data in the exchangeable case. Psychological Bulletin.

[CR35] Hankin BL, Abramson LY (2001). Development of gender differences in depression: an elaborated cognitive vulnerability-transactional stress theory. Psychological Bulletin.

[CR36] Hankin BL, Abramson LY, Moffitt TE, McGee R, Silva PA, Angeli KE (1998). Development of depression from preadolescence to young adulthood: emerging gender differences in a 10-year longitudinal study. Journal of Abnormal Psychology.

[CR37] Hankin B, Stone L, Wright PA (2010). Corumination, interpersonal stress generation, and internalizing symptoms: accumulating effects and transactional influences in a multiwave study of adolescents. Development and Psychopathology.

[CR38] Hartup WW (1989). Social relationships and their developmental significance. American Psychologist.

[CR39] Hartup WW, Laursen B (1993). Adolescents and their friends. Close friendships during adolescence: new directions for child development.

[CR40] Hartup WW (2005). Peer interaction: what causes what?. Journal of Abnormal Child Psychology.

[CR41] Haynie DL (2001). Delinquent peers revisited: does network structure matter?. The American Journal of Sociology.

[CR42] Hibell B, Guttormsson U, Ahlström S, Balakireva O, Bjarnason T, Kokkevi A, Kraus L (2009). The 2007 ESPAD report – substance use among students in 35 European countries.

[CR43] ISTAT (2009). Bilancio demografico nazionale.

[CR44] Jaccard J, Blanton H, Dodge T (2005). Peer influences on risk behavior: an analysis of the effects of a close friend. Developmental Psychology.

[CR45] Johnston LD, O’Malley PM, Bachman JG, Schulenberg JE (2008). Monitoring the future national results on adolescent drug use: overview of key findings, 2007 (NIH Publication No. 08–6418).

[CR46] Joiner TE, Timmons KA, Gotlib IH, Hammen CL (2009). Depression in its interpersonal context. Handbook of depression.

[CR47] Kandel DB (1978). Similarity in real-life adolescent friendship pairs. Journal of Personality and Social Psychology.

[CR48] Katz J, Beach SRH, Joiner TE (1999). Contagious depression in dating couples. Journal of Social and Clinical Psychology.

[CR49] Kiesner J, Poulin F, Nicotra E (2003). Peer relations across contexts: individual-network homophily and network inclusion in and after school. Child Development.

[CR50] Kiuru N, Burk WJ, Laursen B, Salmela-Aro K, Nurmi J-E (2010). Pressure to drink but not to smoke: disentangling selection and socialization in adolescent peer networks and peer groups. Journal of Adolescence.

[CR51] Kiuru, N., Burk, W. J., Laursen, B., Nurmi, J.-E., & Salmela-Aro, K. (2011). Is depression contagious? A test of alternative peer socialization mechanisms of depressive symptoms in adolescent peer networks. *Journal of Adolescence Health*. doi:10.1016/j.jadohealth.2011.06.013. 10.1016/j.jadohealth.2011.06.013 22325130

[CR52] Knecht AB, Burk WJ, Weesie J, Steglich C (2011). Friendship and alcohol use in early adolescence: a multilevel social network approach. Journal of Research on Adolescence.

[CR53] Kuntsche E, Knibbe R, Gmel G, Engels R (2005). Why do young people drink? A review of drinking motives. Clinical Psychology Review.

[CR54] Larsen H, Engels RCME, Granic I, Overbeek G (2009). An experimental study on imitation of alcohol consumption in same-sex dyads. Alcohol and Alcoholism.

[CR55] Larsen H, Overbeek G, Granic I, Engels RCME (2010). Imitation of alcohol consumption in same-sex and other-sex dyads. Alcohol and Alcoholism.

[CR56] Laursen, B., Hafen, C. A., Kerr, M., & Stattin, H. (2011). Friend influence over adolescent problem behaviors as a function of relative peer acceptance: to be liked is to be emulated. *Journal of Abnormal Psychology*. doi:10.1037/a0024707. 10.1037/a0024707 PMC3303164 21823759

[CR57] Markovits H, Benenson J, Dolenszky E (2001). Evidence that children and adolescents have internal models of peer interactions that are gender differentiated. Child Development.

[CR58] Mayeux L, Sandstrom MJ, Cillessen AHN (2008). Is being popular a risky proposition?. Journal of Research on Adolescence.

[CR59] McKenzie DP, Toumbourou JW, Forbes AB, Mackinnon AJ, McMorris BJ, Catalano RF, Patton GC (2011). Predicting future depression in adolescents using the short mood and feelings questionnaire: a two-nation study. Journal of Affective Disorders.

[CR60] McNelles LR, Connolly JA (1999). Intimacy between adolescent friends: age and gender differences in intimate affect and intimate behaviors. Journal of Research on Adolescence.

[CR61] Mercer SH, Derosier ME (2010). Selection and socialization of internalizing problems in middle childhood. Journal of Social and Clinical Psychology.

[CR62] Mercken L, Steglich C, Knibbe R, Vries H (2012). Dynamics of friendship networks and alcohol use in early and mid-adolescence. Journal of Studies on Alcohol and Drugs.

[CR63] Messer SC, Angold A, Costello EJ, Loeber R, Van Kammen W, Stouthamer-Loeber M (1995). Development of a short questionnaire for use in epidemiological studies of depression in children and adolescents: factor composition and structure across development. International Journal of Methods in Psychiatric Research.

[CR64] Newcomb AF, Bagwell CL (1995). Children’s friendship relations: a meta-analytic review. Psychological Bulletin.

[CR65] Organisation for Economic Co–Operation and Development (2009). Education at a glance 2009: OECD indicators. Available from www.oecd.org/edu/eag2009.

[CR66] Overbeek G, Bot SM, Meeus WHJ, Sentse M, Knibbe RA, Engels RCME (2011). Where it’s at! The role of best friends and peer group members in young adults’ alcohol use. Journal of Research on Adolescence.

[CR67] Parker JG, Asher SR (1993). Friendship and friendship quality in middle childhood: links with peer group acceptance and feelings of loneliness and social dissatisfaction. Developmental Psychology.

[CR68] Poelen EAP, Engels RCME, Van Der Vorst H, Scholte RHJ, Vermulst AA (2007). Best friends and alcohol consumption in adolescence: a within-family analysis. Drug and Alcohol Dependence.

[CR69] Popp D, Laursen B, Kerr M, Stattin H, Burk WJ (2008). Modeling homophily over time with an actor-partner interdependence model. Developmental Psychology.

[CR70] Poulin F, Chan A (2010). Friendship stability and change in childhood and adolescence. Development Review.

[CR71] Poulin F, Dishion TJ (2008). Methodological issues in the use of peer sociometric nominations with middle school youths. Social Development.

[CR72] Prinstein MJ (2007). Moderators of peer contagion: a longitudinal examination of depression socialization between adolescents and their best friends. Journal of Clinical Child and Adolescent Psychology.

[CR73] Prinstein MJ & Dodge KA (2008). *Understanding peer**influence in**children and**adolescents*. The Guilford Press.

[CR74] Prinstein MJ, Heilbron N, Guerry JD, Franklin JC, Rancourt D, Simon V (2010). Peer influence and nonsuicidal self-injury: longitudinal results in community and clinically-referred adolescent samples. Journal of Abnormal Child Psychology.

[CR75] Rabaglietti, E., Burk, W. J., & Giletta, M. (2011). Regulatory self-efficacy as a moderator of peer socialization relating to Italian adolescents’ alcohol intoxication. *Social Development*. doi:10.1111/j.1467-9507.2011.00637.x.

[CR76] Raghunathan TE, Rosenthal R, Rubin DB (1996). Comparing correlated but nonoverlapping correlations. Psychological Methods.

[CR77] Ripley R, Snijders TAB & Lopez PP (2011). Manual for SIENA version 4.0. *University of**Oxford*.

[CR78] Rose AJ (2002). Co-rumination in the friendships of girls and boys. Child Development.

[CR79] Rose AJ, Rudolph KD (2006). A review of sex differences in peer relationship processes: potential trade-offs for the emotional and behavioral development of girls and boys. Psychological Bulletin.

[CR80] Rose AJ, Carlson W, Waller EM (2007). Prospective associations of co-rumination with friendship and emotional adjustment: considering the socioemotional trade-offs of co-rumination. Developmental Psychology.

[CR81] Rothon C, Head J, Clark C, Klineberg E, Cattell V, Stansfeld S (2009). The impact of psychological distress on the educational achievement of adolescents at the end of compulsory education. Social Psychiatry and Psychiatric Epidemiology.

[CR82] Rubin KH, Bukowski W, Parker JG, Damon W, Eisenberg N (1998). Peer interactions, relationships, and groups. Handbook of child psychology: social, emotional, and personality development.

[CR83] Rudolph KD (2002). Gender differences in emotional responses to interpersonal stress during adolescence. Journal of Adolescent Health.

[CR84] Schulte MT, Ramo D, Brown SA (2009). Gender differences in factors influencing alcohol use and drinking progression among adolescents. Clinical Psychology Review.

[CR85] Sharabany R, Gershoni R, Hofman JE (1981). Girlfriend, boyfriend: age and sex differences in intimate friedship. Developmental Psychology.

[CR86] Sijtsema JJ, Ojanen T, Veenstra R, Lindenberg S, Hawley PH, Little TD (2010). Forms and functions of aggression in adolescent friendship selection and influence: a longitudinal social network analysis. Social Development.

[CR87] Snijders TAB, Steglich CEG, Schweinberger M, van Montfort K, Oud H, Satorra A (2007). Modeling the co- evolution of networks and behavior. Longitudinal models in the behavioral and related sciences.

[CR88] Snijders TAB, van de Bunt GG, Steglich CEG (2010). Introduction to stochastic actor-based models for network dynamics. Social Networks.

[CR89] Stansfeld SA, Haines MM, Head JA, Bhui K, Viner R, Taylor SJC (2004). Ethnicity, social deprivation and psychological distress in adolescents: school-based epidemiological study in east London. The British Journal of Psychiatry.

[CR90] Starr LR, Davila J (2008). Excessive reassurance seeking, depression, and interpersonal rejection: a meta-analytic review. Journal of Abnormal Psychology.

[CR91] Steglich C, Snijders TAB, Pearson M (2010). Dynamic networks and behavior: separating selection from influence. Sociological Methodology.

[CR92] Steinberg L, Monahan KC (2007). Age differences in resistance to peer influence. Developmental Psychology.

[CR93] Stevens EA, Prinstein MJ (2005). Peer contagion of depressogenic attributional styles among adolescents: a longitudinal study. Journal of Abnormal Child Psychology.

[CR94] Suls J, Green P (2003). Pluralistic ignorance and college student perceptions of gender-specific alcohol norms. Health Psychology.

[CR95] Urberg KA (1992). Locus of peer influence: social crowd and best friend. Journal of Youth and Adolescence.

[CR96] Urberg KA, Degirmencioglu SM, Pilgrim C (1997). Close friend and group influence on adolescent cigarette smoking and alcohol use. Developmental Psychology.

[CR97] Urberg KA, Luo Q, Pilgrim C, Degirmencioglu SM (2003). A two-stage model of peer influence in adolescent substance use: individual and relationship-specific differences in susceptibility to influence. Addictive Behaviors.

[CR98] Van Orden KA, Joiner TE (2006). A role for the contagion of emotion? A comment on Segrin (2004). Journal of Social and Clinical Psychology.

[CR99] Van Zalk MHW, Kerr M, Branje SJT, Stattin H, Meeus WHJ (2010). It takes three: selection, influence, and de-selection processes of depression in adolescent friendship networks. Developmental Psychology.

[CR100] Van Zalk, N., Van Zalk, M. H. W., & Kerr, M. (2011). Socialization of social anxiety in adolescent crowds. *Journal of Abnormal Child Psychology*. doi:10.1007/s10802-011-9533-3. 10.1007/s10802-011-9533-3 21695445

[CR101] Veenstra R, Steglich C, Laursen B, Little TD, Card NA (2012). Actor-based model for network and behavior dynamics: a tool to examine selection and influence processes. Handbook of developmental research methods.

[CR102] Vitaro F, Brendgen M, Tremblay RE (2000). Influence of deviant friends on delinquency: searching for moderator variables. Journal of Abnormal Child Psychology.

[CR103] White HR, Huselid RF, Wilsnack RW, Wilsnack SC (1997). Gender differences in alcohol use during adolescence. Gender and alcohol: individual and social perspectives.

